# Past, present, and future efforts to enhance the efficacy of cord blood hematopoietic cell transplantation

**DOI:** 10.12688/f1000research.20002.1

**Published:** 2019-10-31

**Authors:** Xinxin Huang, Bin Guo, Maegan Capitano, Hal E. Broxmeyer

**Affiliations:** 1 Xuhui Hospital and Institutes of Biomedical Sciences, Fudan University, Shanghai, China; 2 Department of Pathophysiology, Shanghai Jiao Tong University School of Medicine, Shanghai, China; 3 Department of Microbiology and Immunology, Indiana University School of Medicine, Indianapolis, IN, 46202-5181, USA

**Keywords:** Cord Blood, Hematopoietic Cell Transplantation; Hematopoietic Stem Cells; Hematopoietic Progenitor Cells; Regulation of Hematopoiesis; Collection/Processing of Cord Blood; Influence of Oxygen Tension; Ex-vivo Expansion; Homing

## Abstract

Cord blood (CB) has been used as a viable source of hematopoietic stem cells (HSCs) and hematopoietic progenitor cells (HPCs) in over 35,000 clinical hematopoietic cell transplantation (HCT) efforts to treat the same variety of malignant and non-malignant disorders treated by bone marrow (BM) and mobilized peripheral blood (mPB) using HLA-matched or partially HLA-disparate related or unrelated donor cells for adult and children recipients. This review documents the beginning of this clinical effort that started in the 1980’s, the pros and cons of CB HCT compared to BM and mPB HCT, and recent experimental and clinical efforts to enhance the efficacy of CB HCT. These efforts include means for increasing HSC numbers in single CB collections, expanding functional HSCs *ex vivo*, and improving CB HSC homing and engraftment, all with the goal of clinical translation. Concluding remarks highlight the need for phase I/II clinical trials to test the experimental procedures that are described, either alone or in combination.

## Background: the beginning of cord blood transplantation

Cord blood (CB) is a clinical source of transplantable hematopoietic stem cells (HSCs) and hematopoietic progenitor cells (HPCs) for hematopoietic cell transplantation (HCT) ^1– 3^. Until the late 1980’s, the cellular source to treat patients with malignant and non-malignant hematopoietic and other disorders by HCT was mainly bone marrow (BM). Mobilized peripheral blood (mPB), where clinicians utilize known agents (e.g. granulocyte colony-stimulating factor ^4, 5^ and/or HSC/HPC retention signal antagonists such as AMD3100 ^6– 8^) to release high numbers of HSCs and HPCs into the bloodstream for easy collection, was only in its early stages. In the 1980’s, umbilical CB, usually a discarded material except for routine newborn blood tests, was studied for HSC and HPC biology in a national collaboration ^9^. This scientific work was performed at the Indiana University School of Medicine (IUSM). The authors ^9^ demonstrated that there were likely enough HSCs and HPCs for CB clinical transplantation and tha collected cells could be stored for days at room temperature, shipped by overnight express mail to a distant site, and cryopreserved for future CB HCT. The first proof-of-principle CB storage bank for HLA-matched siblings, set up in the Broxmeyer laboratory ^9, 10^, led to the first CB HCT at the Hôpital St. Louis, Paris, in the transplant center directed by Eliane Gluckman as part of an international study ^11^. CB cells were collected and sent from a distant obstetric unit to the Broxmeyer laboratory, where they were tested, cryopreserved, and tested again after thawing of a small separate part of the frozen unit before being hand-delivered to Paris for the clinical HCT. On 6 October 1988, the CB unit was infused into a 6-year-old boy with Fanconi anemia who had been first conditioned by a modified regimen specifically for patients with Fanconi anemia that had been previously developed by Dr. Gluckman, utilizing HLA-matched sibling CB from his sister. This first CB HCT ^11^ was curative for the hematological manifestations of Fanconi anemia; the recipient is alive and well over 31 years later. A total of six more CB HCTs were done in Paris, Baltimore, and Cincinnati using HLA sibling CB cells cryopreserved in the Broxmeyer laboratory to treat Fanconi anemia ^10, 12, 13^ and juvenile chronic myelogenous leukemia (first CB transplant to treat a leukemia) ^14^. Additional information on CB HCT has been reported ^1– 3, 15, 16^ and on the functionality of CB HSCs and HPCs ^17– 30^. Information presented in the original scientific paper was produced years in advance of the clinical transplant, but the scientific ^9^ and clinical ^11^ papers were both published in 1989, as we waited until we knew the first clinical CB HCT ^11^ was successful before submitting the scientific paper ^9^. Cryopreserved CB can be stored for at least 23 and a half years ^18, 31, 32^ with little or no loss of HPCs (comparing thawed cells to pre-freeze numbers). CB HCT was extended to partially HLA-disparate and unrelated donors ^1– 3^. Over 35,000 clinical CB HCT procedures have been performed to date to treat both children and adults with the same malignancies and non-malignancies treated by BM HCT. Advantages of CB HCT are ease of collection and storage of CB without significant risks for the delivering mothers, the ready and quick availability of HLA-typed frozen CB units in public and private CB banks (should such units be rapidly needed for transplantation), and elicitation of relatively low acute and chronic graft versus host disease (GVHD) in recipients after CB HCT, even with unrelated partially HLA-disparate donor cells, compared to that elicited by BM or mPB. Problems include fewer HSCs and HPCs in CB collections than BM or mPB, in part resulting in delayed engraftment of neutrophils, platelets, and immune cells compared to BM and especially with mPB. While not life-threatening, this delay in engraftment with CB prolongs hospital stays, incurring additional health costs ^3, 33^.

Efforts to address slower time to donor blood cell recovery have focused on *ex vivo* (in cell culture) expansion of HSCs (with the idea and possibility that increased numbers of HSCs infused will ameliorate slower time to recovery) or enhancing the homing capacity of HSCs to optimize engraftment. Few such efforts have been tested in the clinical setting ^34– 40^ and only in a few selected transplant centers. This review focuses on enhancing the efficacy of “limited” numbers of HSCs and HPCs in CB collections for CB HCT. A clear distinction must be made between phenotypically recognizable and functional HSCs and HPCs. There are rigorous criteria to phenotypically identify human and mouse HSCs and subsets of HPCs by their cell surface proteins, entailing specific antibodies and flow cytometry. However, phenotype does not necessarily recapitulate functional status. For functional analysis, one must perform specific engraftment studies *in vivo* in mice for mouse and human HSCs and colony forming assays *in vitro* for HPCs ^41, 42^. Recent information on collection, *ex vivo* expansion, and homing of CB HSCs/HPCs for the potential enhancement of CB HCT follows.

## Increasing hematopoietic stem cell numbers in single cord blood collections

Hypoxia is associated with HSC/HPC functions in these cells’ *in vivo* microenvironment ^43^. A means to enhance the efficacy of HCT is through hypoxic collection and processing of HSCs such that the collected cells are never exposed to ambient air oxygen (~21% oxygen) levels ^44, 45^. The BM environment, in which HSCs/HPCs reside, has oxygen levels ranging from 1–5%, with some areas possibly being slightly higher or lower depending on proximity to the vasculature ^46– 49^. Isolating HSCs/HPCs under ambient air (~21% oxygen) exposes these cells to hyperoxic conditions, which within minutes decrease HSC numbers through the differentiation of HSCs to HPCs and not because of HSC cell death ^44, 45^. Studies dating from the 1970’s compared culturing of HSCs and HPCs in low (~5% oxygen), *in vivo* physiological oxygen versus high (~21% oxygen) ambient air oxygen. Culturing human and mouse BM, human CB, and mouse fetal liver at low oxygen *in vitro* increased numbers of detectable functional HSCs/HPCs ^50– 56^. When cultured in low oxygen (48 mmHg, 6.8% oxygen), clonal growth of granulocyte macrophage progenitors (CFU-GM) from mouse BM was enhanced with increased colony numbers and size compared to a more conventional oxygen environment (135 mmHg, 19% oxygen) ^50^. Culturing erythroid progenitors (BFU-E) and more mature erythropoietic precursors (CFU-E) from mouse BM or fetal liver at 5% oxygen increased erythropoietin sensitivity of cells and CFU-E colony numbers ^55^. Human low-density CB cells cultured at 5% oxygen had increased CFU-GM, BFU-E, and multipotential progenitors (CFU-GEMM) and were readily expanded *ex vivo* ^56^. Human BM cultured at 5% oxygen had increased CFU-GM numbers ^51^. Human BM Lin ^–^ CD34 ^+^ CD38 ^–^ cells (enriched for HSCs) cultured at 1.5% oxygen for 4 days had more functional SCID repopulating cells (an assay for functional human HSCs) than comparable human BM cells cultured at 20% oxygen for 4 days (~5.8-fold increase) or freshly isolated Lin ^–^ CD34 ^+^ CD38 ^–^ cells (~4.2-fold increase) ^53^, events associated with stabilization of hypoxia-inducible factor (HIF)-1α, increased surface angiogenic receptors, and VEGF secretion within cultures.

However, in all of these reports, hematopoietic cells were first collected under ambient (~21%) oxygen levels before being placed in culture under lower oxygen and thus the collected cells were already exposed to extra physiological oxygen stress/shock (EPHOSS) or hyperoxia, which induces the production of mitochondrial reactive oxygen species (ROS), increased HSC differentiation, increased functional HPC numbers and cell cycling, and increased mitochondrial mass/activity. EPHOSS effects are mediated by a p53-cyclophilin D-mitochondria permeability transition pore axis and involve HIF-1α and the hypoxamir miR-210 ^45^. Collecting and processing of mouse BM and human CB under low oxygen (3% oxygen, where cells are never exposed to ambient air) resulted in ~two- to five-fold increases in functional HSC numbers (assessed by engraftment in NSG immune-deficient mice) compared to cells collected or processed under ambient air ^44, 45^. Methods to mimic the effects of low oxygen are being examined. Cyclophilin D inhibitor, cyclosporin A (CSA, used to alleviate GVHD in human HCT), resulted in increased mouse BM and human CB HSC numbers and engraftment capability ^45^. However, CSA is difficult to work with. It is hard to get into solution and manifests batch-to-batch variations so that each batch needs to be titrated. Combinations of antioxidants and epigenetic enzyme inhibitors within the flush/collection fluids increased numbers of mouse BM HSCs with increased engrafting capacity in a competitive *in vivo* assay ^57^, but effects of antioxidants and epigenetic enzyme inhibitors have not yet been verified with human CB cells.

## *Ex vivo* expansion of functional hematopoietic stem cells

Small molecules, including, but not limited to, diethylaminobenzaldehyde (DEAB), LG1506, StemRegenin 1 (SR1), UM171, BIO (GSK3β inhibitor), NR-101, trichostatin A (TSA), garcinol (GAR), valproic acid (VPA), copper chelator, tetraethylenepentamine, and nicotinamide, are reported agonists for experimental *ex vivo* expansion of human HSCs and HPCs ^58– 65^. Clinical studies with a few of these small molecules have been reported ^35– 40^. Verification of these clinical studies will take time. SR1 and UM171 are efficient HSC expansion compounds ^58, 61^. SR1, a purine derivative, was identified in a chemical compound screen for candidates promoting *ex vivo* expansion of human HSCs/HPCs ^58^. SR1 binds aryl hydrocarbon receptor and antagonizes AhR signaling in CB HSCs/HPCs, but the exact molecular mechanisms remain unclear. SR1 has been tested in a phase I/II clinical trial ^40^. However, the investigators transplanted both SR1-expanded and -unexpanded CB into patients, so it is too early to determine if SR1-expanded cells contain long-term repopulating HSCs. UM171 promotes *ex vivo* expansion of long-term repopulating HSCs in experimental models ^61^, but the clinical trial using UM171 has not yet been published.

Mechanisms behind mouse and human HSC expansion may be different. Neither SR1 nor UM171 stimulates mouse HSC *ex vivo* expansion ^58, 61^. Thus, mouse studies to evaluate these molecules are not possible. In contrast, overexpression of HOXB4 or co-culturing of recombinant HOXB4 significantly promoted the expansion of both human CD34 ^+^ and mouse HSCs ^66, 67^. Activation of OCT4 was found to enhance *ex vivo* expansion of CB HSCs/HPCs by regulating HOXB4 expression ^68^. Angiopoietin-like proteins support mouse and human HSC expansion in culture ^69^. Overexpression of *MSI2*, an oncogene, antagonizes aryl hydrocarbon receptor signaling and expands human HSCs to levels similar to those seen with SR1 ^70^, even though SR1 does not promote mouse HSC expansion ^71^.

Readout of HSC expansion is related to the culture systems used, which is one reason why the reproducibility of published research may not be easily confirmed. In our experience, serum-free medium such as SFEM (StemSpan ^™^ Serum-Free Expansion Medium, Catalog #09650, Stemcell Technologies) or Stemline (Stemline II HSC expansion medium, Catalog #S0192, Sigma-Aldrich) with 100 ng/mL SCF, TPO, and Flt3L can efficiently maintain CD34 ^+^ HSC and HPC numbers for 7–10 days. Antagonizing retinoid acid receptor (RAR) or PPAR-gamma (PPAR-G) signaling maintains CD34 ^+^ CD38 ^–^ stem and progenitor cell populations when CD34 ^+^ starting cells are cultured in serum and cytokine-containing RPMI-1640 medium, thus facilitating the expansion or maintenance of HSCs in culture ^72, 73^. As SFEM medium is effective in maintaining CD34 ^+^ CD38 ^–^ cell populations, RAR or PPAR-G antagonists do not further enhance the expansion of human HSC production when CD34 ^+^ cells are cultured in SFEM medium. PPAR-G expression was repressed when CD34 ^+^ cells were cultured in SFEM medium ^72, 73^. Most recently, a simple HSC *ex vivo* expansion method was reported by replacing recombinant human serum albumin (HSA) with polyvinyl alcohol (PVA) ^74^. It was suggested that potential contaminants in recombinant proteins might induce inflammatory responses, thus dampening HSC stemness maintenance. By this minor manipulation, the authors reported a massive 900-fold enhancement in functional HSC numbers after 28-day *ex vivo* culture ^74^. This incredibly efficient expansion system needs to be confirmed by other labs. However, such massive increases in functional HSC numbers may not be needed for enhancement of CB HCT. A few fold increase in these cells may suffice to enhance time to engraftment, although the excess expanded cells can be frozen and stored for additional CB transplants.

N ^6^-methyladenosine (m ^6^A) modulates the expression of a group of messenger (m) RNAs critical for stem cell fate decisions by modulating their stability ^75^. Suppressing m ^6^A reader, Ythdf2, which promotes targeted decay of mRNA, promotes *ex vivo* expansion of mouse BM and human CB HSCs ^76^. Conditional knock-out of mouse Ythdf2 increases functional HSC numbers without changing lineage differentiation and without apparent manifestation of hematological malignancies. Knockdown of human YTHDF2 resulted in a 10-fold increase in cytokine-mediated *ex vivo* expansion of human CB HSCs, a 5-fold increase in HPCs, and a greater than 8-fold increase in serial transplantation ^76^. This was associated with enrichment in mRNAs encoding transcription factors in HSCs previously shown to be critical for stem cell renewal. This procedure thus targets multiple effectors rather than one ^76, 77^.

Epigenetic reprogramming using VPA has been utilized to experimentally expand human CB HSCs *ex vivo* ^59^. This required the coordination of cellular reprogramming with remodeling of mitochondria and activation of p53 that apparently limits ROS levels ^78^, which induce HSC differentiation ^44, 45^. DEK, a nuclear heterochromatin remodeling agent, which can be secreted from the cell and act as a cytokine that manifests its effects through the chemokine receptor CXCR2 (the only known non-chemokine to bind CXCR2), enhances cytokine-mediated *ex vivo* expansion of human CB and mouse BM HSCs and HPCs ^79^.

One challenge for *ex vivo* expansion is the lack of markers labeling functional HSCs during and after *ex vivo* culture. Some signaling pathways might stimulate the expansion of CD34 ^+^ cells, most of which are progenitors. Markers including CD90 or CD49f have often been used to isolate HSCs from fresh human CB and BM samples. However, CD34 ^+^ CD90 ^+^ CD49f ^+^ phenotypic HSCs do not necessarily reflect functional HSCs, especially under stress conditions such as *ex vivo* expansion ^41^. The only currently available way to confirm the expansion of human HSC numbers is through transplantation using sublethally irradiated immune-deficient mice. A logistical problem is that *ex vivo* clinical studies will likely have to be performed in very select centers with expertise for these procedures. Also, the economics associated with *ex vivo* expansion must be taken into account, as it will likely add significant additional costs to the clinical HCT procedure. While it does not appear that *ex vivo* expansion procedures have damaged HSCs or caused pre-leukemia/leukemia, anytime HSCs are manipulated *ex vivo*, there is potential for long-term detrimental effects, which may involve gene expression pattern changes and epigenetic modifications that might result in long-term counter-productive outcomes.

During *ex vivo* expansion, we must also keep in mind the different physical characteristics of the *in vivo* HSC BM niche that help maintain HSC homeostasis (e.g. interactions that HSCs have with the other cells within their BM niche and lower oxygen concentrations within the BM). Taking these factors into account, CB HSCs/HPCs have been expanded in the presence of mesenchymal stem/stromal cells and have been proven to be safe in a clinical study ^36^. In addition, hypoxia culturing (5% oxygen) after cells were collected in ambient air potentiated *ex vivo* expansion of CB HSCs/HPCs ^80^.

Although *ex vivo* expansion is a promising means to overcome limited numbers of CB HSCs collected for transplantation, there is still much work to be done in this area. More mechanistic insight is required regarding the regulation of HSC stemness.

## Improving hematopoietic stem cell homing to enhance cord blood hematopoietic cell transplantation

After infusion into peripheral blood, HSCs home to the BM microenvironment by sensing chemical gradients of chemoattractants ^81^. The BM microenvironment provides a unique matrix bedding and conducive signaling environment supporting long-term engraftment and balances HSC proliferation and differentiation ^82, 83^. HSC homing is crucial for successful clinic outcomes ^84^.

Directing HSC migration and homing from the peripheral circulation to the BM involves interactions between chemokine ligand CXCL12/stromal cell-derived factor (SDF)-1 and its receptor CXCR4 ^85, 86^. CXCL12 is highly expressed by BM stromal cells padding the stem cell niche. Gradients of CXCL12 provide directional cues and orchestrate HSC migration towards the BM. CXCR4 is a seven-transmembrane G-protein-coupled chemokine receptor expressed on the surface of HSCs. Knockouts of CXCL12 or CXCR4 result in severe hematopoietic defects ^87– 89^. Sphingosine-1-phosphate (S1P) and ceramide-1-phosphate (C1P) also provide homing gradients guiding HSCs to BM niches ^90– 92^. Strategies to enhance HSC homing are classified into three categories: regulation from the cell membrane, regulation in the cytoplasm, and regulation in the nucleus.

### Regulation from the cell membrane

The cell membrane is a semi-permeable membrane separating the cell from the external environment. The cell membrane consists of phospholipid bilayers and many membrane-associated proteins, while lipids are fundamental structural elements, and proteins are responsible for performing specific membrane functions ^93, 94^. Lipid rafts are special membrane domains rich in glycosphingolipids and cholesterol and have been implicated in regulating membrane signaling ^95, 96^. Incorporation of CXCR4 into lipid rafts facilitates the sensing of CXCL12 gradients, enhancing homing and engraftment of HSCs ^97, 98^. Short-term (4-hour) mild heating (39°C) led to elevated membrane lipid raft formation, resulting in increased CXCR4 aggregation and co-localization with lipid rafts, and promoted human CB HSC homing and engraftment in an NSG mouse transplantation model ^99^. One report showed a beneficial effect of dimethyl sulfoxide (DMSO) treatment on HSC homing, possibly because of lower internalization of the surface CXCR4 receptor ^100^.

Another HSC homing regulator found on the cell membrane is dipeptidyl peptidase 4 (DPP4). DPP4, also referred to as cell surface CD26, a 110 kDa serine protease. It cleaves penultimate alanine or proline amino acids at the N-terminus of target substrates including cytokines and chemokines ^101, 102^. DPP4 is widely expressed in tissues, e.g. liver, spleen, lung, and BM, as a membrane-bound form and is also found in serum in soluble form. DPP4 is expressed on the surface of HSCs and HPCs, as well as on T lymphocytes; it is an important regulator of HSC and T cell function ^101– 103^ and modulates HSC homing at least in part by modifying CXCL12 ^104, 105^. DPP4 generates a truncated form of CXCL12, which is no longer chemotactic but is able to block chemotaxis of full-length CXCL12 ^104^. Blocking enzymatic activity of DPP4 increases levels of non-truncated CXCL12, enhancing HSC homing and engraftment of human CB CD34 ^+^ cells or mouse BM cells ^105, 106^. Sitagliptin, an FDA-approved orally active inhibitor of DPP4, has been used to enhance the engraftment of single CB transplantation in patients with leukemia and lymphoma ^107– 109^. Since it was subsequently realized that DPP4 truncated a number of other hematopoietic-regulating cytokines ^110^, the time to engraftment may have been further decreased had sitagliptin been given over more days.

Prostaglandin E2 (PGE _2_) is an important mediator of physiological and pathological systems ^111, 112^. Pulse treatment of human and murine HSCs with PGE _2_ results in enhanced HSC homing and engraftment, mediated through the upregulation of surface CXCR4 levels ^22^. A clinical trial evaluating the effects of PGE _2_ in CB HCT has elicited promising results, with apparently faster neutrophil recovery and long-term dominance of the PGE _2_-treated CB unit ^34^, but this study was done using PGE _2_-treated and -untreated CB. The long-term engrafting capability of the PGE _2_-treated cells is unknown. PGE _2_ has four specific G-protein-coupled receptors on the cell membrane, EP1–EP4 ^113^. EP2 and EP4 were involved in the upregulation of CXCR4 and CXCL12 expression and promoted HSC migration towards CXCL12 ^114^. It may be practical to develop better EP2 and EP4 agonists to further enhance HSC homing and engraftment. In an animal model, combining PGE _2_ treatment of donor cells and *in vivo* DPP4 inhibitor demonstrated additive effects on enhancing mouse BM engraftment into lethally irradiated mice ^115^, suggesting potential enhancement in efficacy by combining two different treatment modalities.

Calcium-sensing receptor (CaR) is a cell membrane G-protein-coupled receptor mediating cell responses to extracellular calcium ^116^. CaR knockout HSCs are defective in adherence to the BM microenvironment and fail to engraft after transplantation ^117^. Treatment of murine HSCs with a CaR agonist, cinacalcet, led to enhanced HSC homing and engraftment, effects mediated through intracellular CXCR4 signaling ^117^. CXCR4 mRNA and surface expression remained unaltered, so cinacalcet may stimulate enhanced CXCR4 signaling in an unconventional manner.

During the homing process, the first early step is considered to be HSCs rolling on P-selectins and E-selectins of endothelial cells in BM ^30^. P-selectins and E-selectins are C-type lectins whose ligands must be properly α1,3-fucosylated to form mature glycan determinants. Increasing the levels of cell surface fucosylation has been shown to enhance the engraftment of CB cells in immunodeficient mice ^30, 118, 119^. Furthermore, CB units were treated with guanosine diphosphate fucose and fucosyltransferase-VI to enhance cell surface fucosylation in a clinical trial, and the results showed improved engraftment efficiency of fucosylated cells ^27^.

### Regulation in the cytoplasm

Heme oxygenase 1 (HO-1), an endoplasmic reticulum (ER)-anchored enzyme, plays important roles in anti-oxidative and inflammatory processes ^120^. HO-1 acts as a negative regulator of HSC homing. HO-1 knockout HSCs have enhanced migration towards CXCL12 and S1P gradients ^121^. Transient treatment with HO-1 inhibitor (SnPP) increased chemotaxis and homing of HSCs/HPCs ^121^.

### Regulation in the nucleus

The glucocorticoid receptor (GR) is an evolutionarily conserved nuclear receptor to which glucocorticoids bind ^122, 123^. Upon ligand binding, GR is transported into the nucleus and functions as a transcriptional factor to activate downstream gene transcription, regulating numerous physiological processes. Glucocorticoid treatment of human CB HSCs significantly elevated surface CXCR4 expression and increased chemotaxis towards CXCL12, HSC homing, and engraftment in NSG mice ^123^. Activated GR transfers into the nucleus and binds to glucocorticoid response elements in the CXCR4 promoter in human CB HSCs, followed by recruitment of SRC1/p300 histone acetyltransferase complex. This promotes histone H4K5 and H4K16 acetylation in the CXCR4 promoter region, leading to upregulation of CXCR4 transcription. Knockdown of SRC1 or p300 suppresses the effects of activated GR on CXCR4 surface expression, while inhibition of p300 by a small molecule inhibitor, C646, blocks the enhanced homing effects of activated GR ^72^, suggesting that activated GR depends on histone acetylation to promote HSC homing.

Histone deacetylases (HDACs) are crucial modulators in regulating histone acetylation levels ^124, 125^. HDACs remove acetyl groups from lysines of target proteins and play important roles in physiological processes ^124^. The treatment of human CB HSCs with HDAC inhibitors substantially increased surface CXCR4 expression, improved chemotaxis towards CXCL12, and enhanced HSC homing and engraftment ^126^. There are 18 HDAC enzymes in mammals, grouped into five subfamilies based on sequence similarity (class I, IIa, IIb, III, and IV) ^127^. HDAC5 is the one HDAC specifically involved in the regulation of CXCR4 expression and HSC homing ^128^. HDAC5 inhibition increased acetylation levels of histones at the CXCR4 promoter region as well as p65 acetylation levels in the nucleus. NF-κB subunit p65 is a crucial transcription factor regulating CXCR4 expression. The acetylation of p65 enhances its DNA-binding activity and promotes target gene transcription ^127– 130^. Blocking NF-κB signaling suppressed the effects of HDAC5 inhibition on CXCR4 upregulation and enhanced HSC homing ^126, 128^, indicating essential roles for NF-κB signaling in regulating HSC homing and demonstrating a previously unknown negative regulation of HSC homing by HDAC5.

HIF-1α, a DNA-binding transcriptional factor, mediates cellular responses to hypoxia ^43, 129^. HIF-1α is important during animal development and for energy metabolism. The BM microenvironment, where HSCs reside, is hypoxic ^43^. HIF-1α is stabilized in HSCs and regulates HSC activity/quiescence ^131, 132^. Pharmacological increases in HIF-1α promote HSC homing and engraftment also by upregulating surface CXCR4 expression ^133^. CXCR4 expression upregulation results from HIF-1α binding with hypoxia response elements, located at –1.3 kb from the transcription start site of the CXCR4 promoter region. Caffeic acid phenethyl ester (CAPE) treatment promotes HSC homing and engraftment by inducing the expression of HIF-1α. CAPE administration upregulates HIF-1α protein levels and CXCL12 in BM endothelial cells and inhibition of HIF-1α by PX-478 suppresses CAPE-mediated enhanced HSC homing, further supporting the notion that HIF-1α is important during HSC homing and engraftment.

The above-mentioned approaches for homing range from the cell membrane (lipid rafts, DPP4, EP2 and EP4, and CaR) to the cytoplasm (HO-1) and inside the nucleus (GR, HDAC5, and HIF-1α). Which procedure would be the best to be tested in a clinical setting needs to be determined. Perhaps combinations of approaches can further increase the homing and engraftment of HSCs. It may be that short-term treatment of donor CB units for about 16 hours may provide significant enhancement for engraftment in the setting of CB HCT, possibly negating the necessity for *ex vivo* expansion efforts. Alternatively, it may be that the CB cells do not have to be pretreated *ex vivo* prior to infusion into the patient but rather that the cells can be infused into the patient who is then given the reagents *in vivo* to enhance the homing/engrafting capability of the infused cells. It is also possible that *ex vivo* expanded HSCs may better engraft if it turns out that homing of expanded HSCs is suboptimal and can be enhanced.

## Concluding remarks

Enhancing CB HCT efficacy will not only reduce the time of donor cell recovery but also make it possible to use more banked CB units that contain fewer HSCs/HPCs. There are a number of new ways to potentially enhance the efficacy of CB HCT ^134^. However, most are laboratory efforts. How to get these new methods into clinical trials is a problem that needs to be solved. We believe that simpler is always better. The simpler the procedure, the more likely that it will be clinically translated. There are just not enough clinical CB HCTs available to set up phase I/II clinical trials to test these new procedures. Most investigators doing such trials are “wed” to their personal favorite procedure. If, in the future, we can deal with this problem and find means for additional clinical efforts, it is possible that several new procedures can be used together ^134^. This, however, adds additional logistical problems versus the use of one procedure. Clinical trials are costly, and it is not clear where the money to pursue such trials will come from, or even if they can be supported at all, since current trials are funded by companies to test their own products. A gathering of interested scientists and clinical investigators who can think-tank this problem is desperately needed and strongly encouraged.
